# Wound healing properties and antimicrobial activity of platelet-derived biomaterials

**DOI:** 10.1038/s41598-020-57559-w

**Published:** 2020-01-23

**Authors:** Aref Shariati, Alireza Moradabadi, Taher Azimi, Ehsanollah Ghaznavi-Rad

**Affiliations:** 1 Department of Microbiology, School of Medicine, Iran University of Medical Sciences, Tehran, Iran; 2 Department of Microbiology & Immunology, Faculty of Medicine, Arak University of Medical Sciences, Arak, Iran; 3 Molecular and Medicine Research Center, Faculty of Medicine, Arak University of Medical Sciences, Arak, Iran; 4 Department of Pathobiology, School of Public Health, Tehran University of Medical Science, Tehran, Iran

**Keywords:** Cell biology, Immunology, Microbiology, Pathogenesis

## Abstract

We analyzed the potential antibacterial effects of two different PdB against methicillin-resistant *S. aureus* and *P. aeruginosa*. The third-degree burn wound healing effects of PdB was also studied. Blood samples were obtained from 10 healthy volunteers and biological assays of the PdB were performed and the antimicrobial activity against MRSA and *P. aeruginosa* was determined using disk diffusion (DD), broth microdilution (BMD), and time-kill assay methods. 48 Wistar albino rats were burned and infected with MRSA. Two groups were injected PdB, the control groups were treated with plasma and received no treatment respectively. In the next step, the rats were euthanized and skin biopsies were collected and histopathologic changes were examined. The results of DD and BMD showed that both PdB performed very well on MRSA, whereas *P. aeruginosa* was only inhibited by F-PdB and was less susceptible than MRSA to PdBs. The time-kill assay also showed that F-PdB has an antibacterial effect at 4 hours for two strains. Histopathological studies showed that the treated groups had less inflammatory cells and necrotic tissues. Our data suggest that PdB may possess a clinical utility as a novel topical antimicrobial and wound healing agent for infected burn wounds.

## Introduction

Among the traumatic injuries, burns are the main factor of mortality that causes structural and functional deficiencies in numerous organ systems. So today, despite many advances in infection control and wound healing, burn wound infection causes 60% of deaths in burned patients and 300,000 deaths worldwide each year^1,2^. Burns damage skin integrity and weaken cellular and humoral immunity. Necrotic tissues also reduce the presence of immune cells in the burned area and facilitate the penetration of pathogens into the underlying tissues and promote their spreading^3,4^. Burn wounds are highly susceptible to infection due to the issues mentioned above. Therefore, as the burn sites are colonized with microorganism the burn wound invasive infections such as sepsis may occur. Methicillin-resistant *S. aureus* (MRSA) among gram-positive and *Pseudomonas aeruginosa* (*P. aeruginosa*) among gram-negative bacteria are predominant pathogens in burn wound infection^5,6^. *S. aureus* invades eschar, penetrates subcutaneous and unburned tissues and may create abscesses with different sizes. These abscesses protect *S. aureus* against host immunity and antibiotics and provide the conditions for entry of this bacterium into the bloodstream^7^. The subsequent development and use of broad-spectrum antibiotics effective against *S. aureus* resulted in the emergence of gram-negative organisms, particularly *P. aeruginosa*, as the predominant organisms causing invasive burn wound infections in burn patients^8,9^. It is worth noting that, the intrinsic and acquired properties such as efflux pumps and reduction of the permeability of the outer membrane proteins have created a high-level antibiotic resistance in *P. aeruginosa*, which has limited the use of antibiotics like fluoroquinolones, β-lactams, and carbapenems to control burn wound infections^10,11^. platelets are cells that play an important role in primary coagulation. However, platelets also absorb monocytes and macrophages after activation, therefore; platelets also play a role in secondary immunity^12,13^. Platelet granules contain antimicrobial peptides and growth factors such as platelet-derived growth factor, transforming growth factor (TGF), vascular endothelial growth factor (VEGF) and insulin-like growth factor (IGF) that affect the migration and binding of immune cells^14,15^. Furthermore, platelets cause skin cell regeneration, angiogenesis, collagen, and fibroblastic production that makes the platelets as a good candidate for wound healing^16^. But, due to immunologic reactions, the use of platelets has been restricted^16^. Platelets can be easily separated from the blood by centrifugation. Then, their granule content can be released by calcium chloride or physical methods group such as freeze-unfreeze technique. Therefore, in this case not only the immunological responses to the platelets are prevented, but also the antimicrobial activity and wound healing process are enhanced^17,18^. Therefore, the purpose of present study was to evaluate the effect of platelet derived biomaterials as a blood-extracted biomaterial on *P. aeruginosa* and MRSA in order to find a new strategy for inhibiting these bacteria and burn wound healing.

## Result

### Antimicrobial susceptibility

The results of the Kirby-Bauer disk diffusion (DD) method showed that the F-PdB had a good inhibitory effect on MRSA (1 mm), while the CaCl_2_-PdB had a less inhibitory effect (0.5 mm). On the other hand, results showed that none of the PdBs were able to inhibit *P. aeruginosa* growth and no inhibition zone was observed for this bacterium. Due to the lack of proper distribution of the PdB in DD, broth microdilution methods were used and CaCl_2_-PdB and F-PdB were prepared in different dilutions (ranging from 1:2 to 1:2048) in wells. Both CaCl_2_-PdB and F-PdB were able to inhibit the growth of MRSA at the dilution of 1:2. Furthermore, F-PdB had an inhibitory effect on *P. aeruginosa* at its highest concentration (1:2), while CaCl_2_-PdB could not inhibit bacterial growth. It should be noted that the PdBs could be distributed in the microdilution method more than the DD and furthermore, the acidity and pH can also affect the antimicrobial activity of PdBs^19^, the inhibitory effect on *P. aeruginosa* was found in this method as opposed to the DD. Control wells (Plasma, CaCl_2_, and PBS) did not show any inhibitory effect on the tested concentrations, and no growths were observed in wells containing merely CaCl_2_-PdB, F-PdB and, culture medium. This indicated that the PdBs were not contaminated.

### Time-kill assay

**Figure 1:**
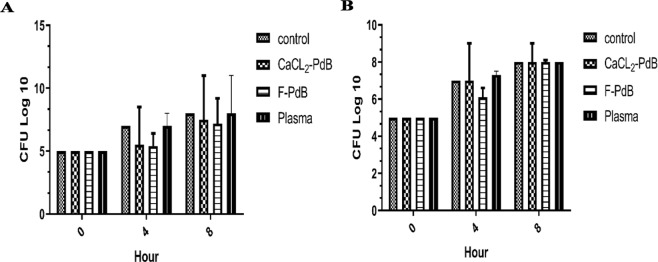
Antimicrobial activity of F and CaCl_2_-PdB against (**A**) MRSA and (**B**) *P. aeruginosa* in time-kill assay method compared with the control (initial inoculums at time 0).

The results of this method showed that both CaCl_2_-PdB and F-PdB were able to reduce the number of MRSA bacteria compared to the control group. The highest decrease in the number of colonies was observed in the first 4 hours (1.5 Log 10) and there was no significant difference between the two PdB groups (P < 0.05). By contrast, *P. aeruginosa* was less susceptible to PdB than the MRSA and the bacterial population was reduced only by the F-PdB (0.4 log10), whereas CaCl_2_-PdB did not inhibit the growth of *P. aeruginosa*. As the bacteria increased in number after 8 hours, again, none of the extracts showed a bactericidal effect. Since the inoculums were reduced between 0 and 3 log10 CFU/ml (Fig. 1), they showed a bacteriostatic effect^20^.

### CaCl_2_-PdB and F-PdB effects on the bacterial burden in MRSA infected burn wounds

**Figure 2:**
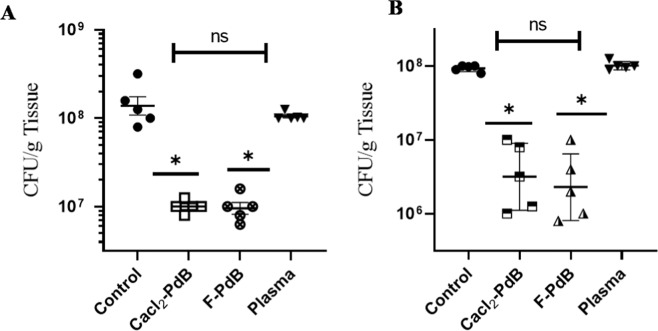
Wound bacterial burden on day 3 (**A**) and day 7 (**B**) in mice infected intradermally with 5 × 10^8^ cells MRSA was determined by the amount of CFU growth (n = 5 wounds per group). The bacterial burden of plasma, CaCl_2_-PdB, and F-PdB treated wounds in both bacteria were significantly lower than untreated, but no significant differences were observed between two the PdBs. Error bars denote SEM. *p ≤ 0.05, ns p > 0.05 (CFU; colony forming unit).

The *in vivo* evaluation of antimicrobial properties of CaCl_2_-PdB and F-PdB against MRSA was performed by homogenizing infected burn wounds and quantifying the present CFUs in tissue on days 3 and 7 (Fig. 2A,B, respectively). The wounds treated by CaCl_2_-PdB and F-PdB significantly showed a decrease in bacterial counts compared to the control group (infected, untreated group) (p ≤ 0.05). However, CaCl_2_-PdB and F-PdB antimicrobial effects did not differ significantly from each other (p > 0.05). Both of the PdBs decreased the number of bacteria on days 3 and 7 compared to the control group (which did not receive any treatment).

### Histological evaluation of wound healing

**Figure 3:**
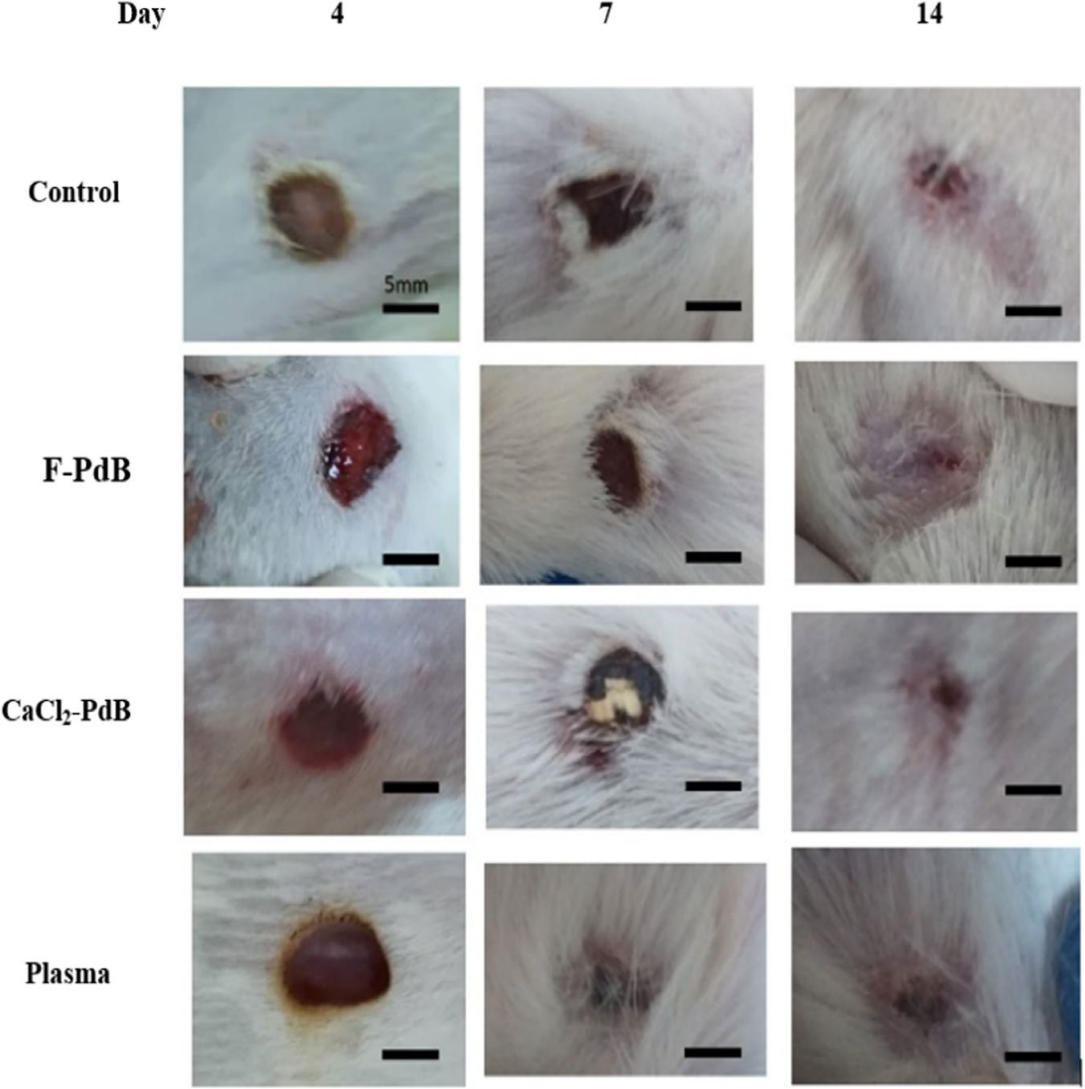
Representative images of wound healing from days 1–14. Subcutaneously administration of F-PdB and CaCl_2_-PdB decreased eschar size and qualitatively accelerated healing compared to control groups (infected control and plasma treated). Scale bar = 5 mm.

**Figure 4:**
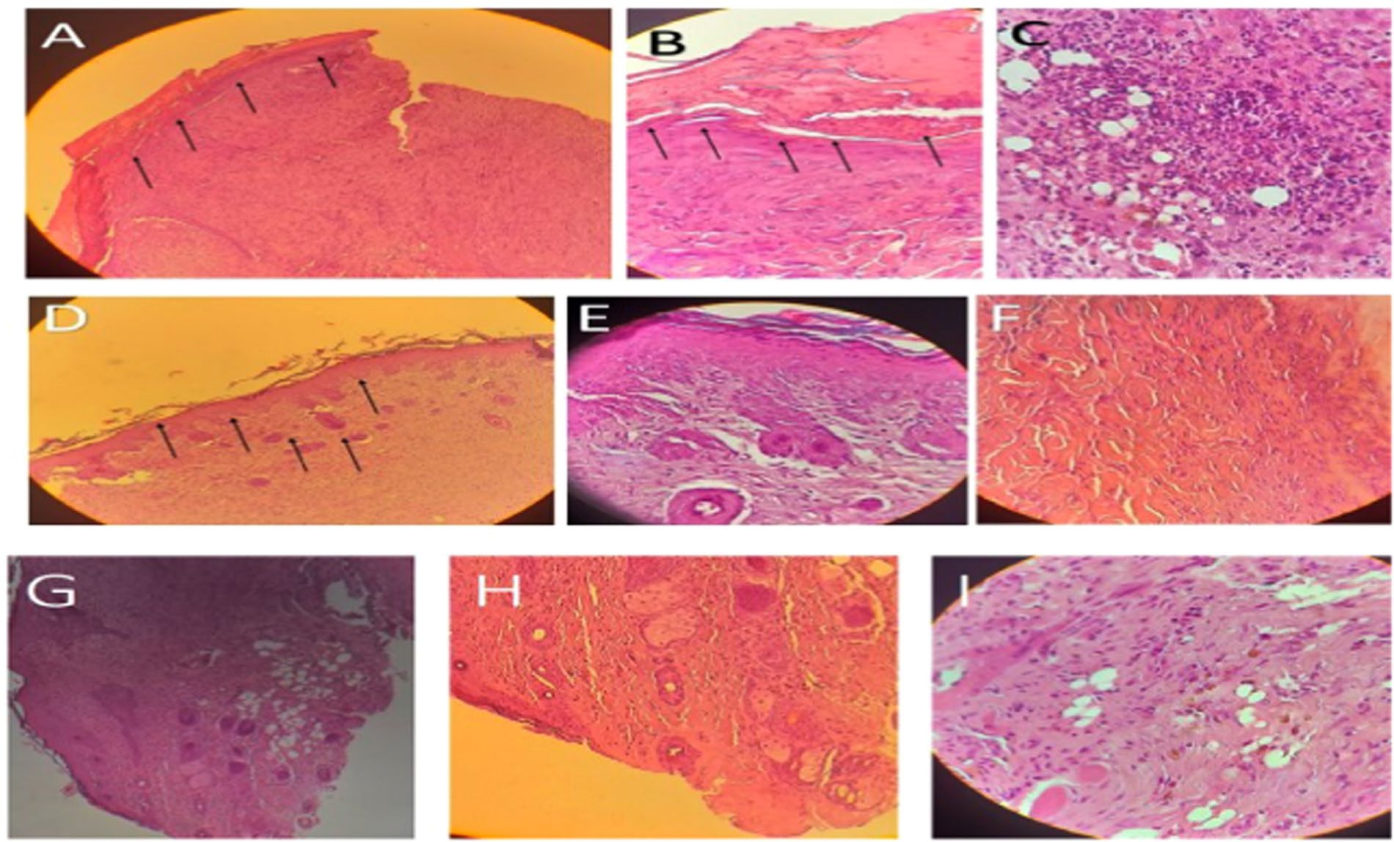
Histological analysis of wound tissue from day 14 using hematoxylin and eosin (H&E) (magnification 10× , and 40×). (**A**,**D** and **G**) compared the epidermis and the extracellular matrix which is thinner in (**A**) (untreated control wounds) compared to (**D** and **G**) (freeze or CaCl_2_-PdB treated wound). (**B**,**E** and **H**) 40× magnification of the Histologic wound shows the separated epidermis in B (untreated control wounds) and new angiogenesis in (**E** and **H**) (freeze or CaCl_2_-PdB treated wound). (**C**,**F** and **I**) 40× magnification of the Histologic wound shows the inflammation and cell aggregation in (**C**) (untreated control wounds) and collagen production (**F** and **I**) (freeze or CaCl_2_-PdB treated wound).

**Figure 5:**
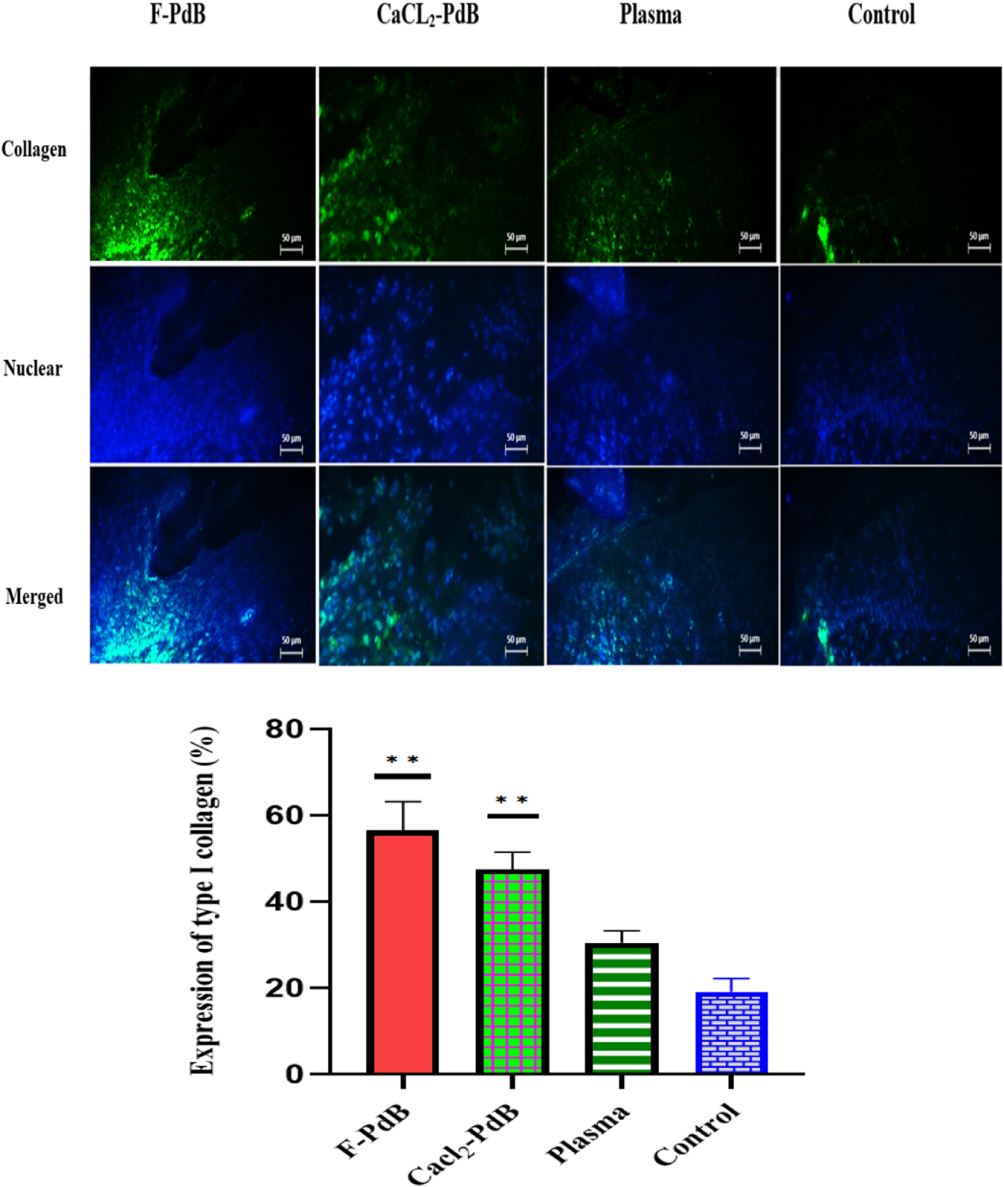
Fluorescent staining of collagen type I. The wound collagenization rate of rat skin samples which were immunolabelled with the antibody against collagen type I was measured on day 14. Wounds treated with F-PdB and CaCl_2_ showed greater collagen deposition and organization compared to the control groups (P < 0.05). The blue fluorescence represents DAPI nuclear-staining which confirms the presence of the cells. The percentages of the cell or tissue visual expression were measured using image j software. Scale bars are 50 µm. The statistical significance was measured by the Kruskal Wallis test and data are presented as mean ± SEM.

In comparison to the control group, the treated group demonstrated a more accelerated wound healing process in the rats (Fig. 3). Moreover, the qualitative assessment showed that in the wounds treated by F-PdB and CaCl_2_-PdB the number of inflammatory cells was reduced. This phenomenon causes new angiogenesis, higher collagen deposition and reepithelization earlier in the treated groups than the control group (Fig. 4). Histopathologic examinations on specimens obtained from the rats of different groups on day 14 showed that the PdBs treated groups had a better wound healing than the control group that received no treatment and the rats treated with plasma. It is noteworthy that both PdBs accelerated maturation and formation of the epidermis compared to the control group which has thinner neo-epidermis, whereas inflammatory granulation tissue, inflammation, and cell aggregation were also present in smaller amounts in PdB-treated rats. An increased amount of collagen and a reduction in necrotic tissue caused by burns were also observed in the PdB-treated groups. It should be noted that greater collagen deposition and organization were seen in the groups treated with F-PdB and CaCl_2_-PdB than in the two control groups, although there was no significant difference between the two treatment groups (Fig. 5). Furthermore, an increase in angiogenesis that indicates the proliferative phase of healing was observed in the F and CaCl_2_-PdB treated groups. It should be noted that the plasma-treated group did not undergo platelets activation and was used as the control. The results showed that plasma did not have any effect on reducing the number of MRSA and wound healing. Besides, there was no difference in collagen production, reduction of inflammatory cells and angiogenesis between the F-PdB and CaCl_2_-PdB treated groups.

## Discussion

The mechanism by which the platelets inhibit the growth of microorganisms, reduce skin infections and enhance wound healing still remains elusive. Therefore, scientific studies about the applications of these preparations are relatively scarce^21^.Various factors such as platelets antimicrobial peptides (e.g. RANTES), myeloperoxidase, platelets factor-4, connective tissue activating peptide III, platelet α-granules components and the high role of platelets in innate immunity are major factors inhibiting the growth of microorganisms^21,22^. Very few studies have evaluated both antimicrobial and wound healing properties of a PdB, so this has limited the clinical translatability of those findings. Therefore, in the currents study, two different methods were used to activate platelets (CaCl_2_-PdB and F-PdB) and investigated their antimicrobial activity; in addition, these biomaterials were also tested for the healing of burn wounds. *S. aureus* and *P. aeruginosa*, two of the major pathogens isolated from burn wound infections, readily reach the bloodstream due to their different virulence factors and cause the death of the patient after septicemia. Furthermore, due to the emergence of antibiotic resistance, today, control of these bacteria has become very important^10^. The results of our DD and MIC methods showed that the PdB performed very well on MRSA but the results were different for *P. aeruginosa* and only F-PdB had an inhibitory effect on this bacterium. The reason for the differences in the sensitivity of different microorganisms to platelets antimicrobial peptides is not well understood, but it seems that the phenotypic difference of infecting bacteria is one of the main factors^23^. Previous studies have suggested that decreased sensitivity of *S. aureus* to antimicrobial agents of platelets may be related to reducing transmembrane potential, mutations in genes that encode proteins involved in the generation of the proton motive force or an increase in membrane fluidity^20,24^. Nevertheless, it seems that the more resistance of *P. aeruginosa* compared to *S. aureus* is further depended on differences in cell membrane charge and composition that reduces the interaction of platelet antimicrobial agents with the cell surface of gram-negative bacteria^25^. However, specialized methods such as bacterial cell surface imaging using electron microscopy after interaction with platelets are needed for a better understanding of these mechanisms and it may be possible to overcome cell membrane charge by using different ways of activating Platelets. This has demonstrated well in *Aniytau et al*. study that has investigated the effect of plasma rich in growth factors obtained in 5 different formulations on methicillin-resistant and susceptible *S. aureus* and *S. epidermidis*. The results of their study showed that unlike the other strains, only methicillin-sensitive *S. epidermidis* were not well inhibited by plasma rich in growth factors while there was no plausible reason for this^20^. In another investigation, the antimicrobial effect of platelet-poor plasma, platelet-rich plasma, platelets gel, and solvent/detergent-treated platelets lysate from two donors on various gram-positive and gram-negative pathogens showed that *Escherichia coli* was inhibited well, but none of the plasma and platelets materials showed antimicrobial activity against *Enterobacter cloacae*, *Bacillus cereus*, *Bacillus subtilis*, and *S. epidermidis*^21^. Previous studies have shown that different platelets factors such as platelets-derived growth factors (PDGFs), TGF, VEGF, and IGF have an important role in the proliferation and survival of mesenchymal cell lineages and tissue repair. So, the present study aimed to evaluate the role of PdB in burn wound healing^26,27^. In addition to evaluate the antimicrobial activity, our results showed that PdB can also heal burn wounds. Collagen production, decreased necrotic tissue, re-epithelialization, and increased neovascularization was seen more in the PdB-treated groups compared with the control group. When platelets are activated by CaCl2 or freeze/unfreeze method, degranulation of platelet alpha granules occurs and growth factors release^28,29^. Furthermore, the platelet-derived endothelial cell growth factor is a stimulant for the collagen deposition also a chemotactic factor for macrophage, neutrophils, and monocytes^29^. Moreover, this factor regulates the wound-healing process by secreting additional growth factors, such as tumor necrosis factor-a (TNF-a), basic fibroblast growth factor (bFGF) and triggers vessel proliferation by VEGF, PDGF, and bFGF^30,31^. Therefore, the better antimicrobial performance of PdB in the *in vivo* than *in vitro* conditions relies on the high impact of PdB on the intense inflammatory response, release of soluble mediators, homing of circulating cells to damaged tissues which control infection, and wound healing^26^. *Henderson* and his colleagues reported that autologous platelet gel can heal wounds in pigs by the production of the extracellular matrix, fibroblastic proliferation, and collagen^32^. *Umit Ozcelik et al*. reported that PRP reduced inflammatory cell infiltration and had an effective role in burn healing after partial-thickness burn injury, which could be used in future studies for human wounds^15^. But they reported that PRP had no effect on fibroblast development, collagen production, vessel proliferation, or epithelization. The results of another study showed that PRP accelerated wound closure and reduced the number of inflammatory cells in deep second-degree burns and deep second-degree burns associated with diabetes mellitus but not in the third-degree burns^33^. Previous studies have used various approaches, including the use of nanoparticles to control burn wound infections^25,34^. These substances can have side effects on the eukaryotic cells, so the use of nanoparticles is limited whereas no side effects have been reported for platelet biomaterials. Besides, PdBs accelerate tissue healing and improve the efficiency of tissue repair following injury in a safe, reliable, and economical manner^25,34^. In the present study, the antibacterial effect of PdB on MRSA and *P. aeruginosa* was investigated furthermore *in vitro* and *in vivo* effects were compared. Due to the good healing effect of PdB on burn wounds, this product can be used to make wound dressings to form a barrier against fluid loss and microorganism contaminations. Moreover, it improves wound healing by increasing the cells in the area, and allows vessel proliferation, keratinocyte adhesion, and differentiation. Finally, it should be noted that a wide range of microorganisms, including bacteria (coagulase-negative staphylococci, *Enterococcus* spp, *Klebsiella pneumoniae*, *Serratia marcescens*, *Enterobacter* spp. *Proteus* spp. and *Acinetobacter* spp), fungi (*Candida* spp., *Aspergillus* spp., *Fusarium* spp.) and viruses (herpes simplex virus, cytomegalovirus, varicella-zoster virus), are effective in burn wound infections, and the antimicrobial activity of PdBs on these microorganisms also should be assessed.

## Methods and Materials

### Ethics statements

All experimental protocols and procedures for animal experimentation (rats) were carried out in accordance with relevant guidelines and regulations and were approved by the Ethics Committee on Animal Research of Arak University of Medical Science (protocol number IR.ARAKMU.REC.1397.275).

### Platelet-rich plasma and plasma-derived biomaterial preparation

In this study, for preparing platelet-rich plasma (PRP) the expired human platelet products were used. All methods involving human blood products obtained from a blood bank were carried out in accordance with relevant guidelines and regulations. It should be noted that for the preparation of PdBs, we used expired human platelet products, so there was no ethical problem. 50cc of whole blood (blood and sodium citrate) was centrifuged at 1000 g for 10 minutes. The plasma rich fragment (PRF) was obtained from the supernatant of whole blood and was prepared as a blood product in the blood bank center. For enrichment of the PRF, it was centrifuged at 1500 rpm for 15 minutes to precipitate the platelets. The platelet-poor plasma (PPP) was prepared by separating the supernatant and pipetting it in a separate tube. PRP, on the other hand; remained in the centrifuged tube with the platelet concentration of 2500000 per microliter. During the preparation of PRP, samples were separated using Pasteur pipette and the count of platelet in PRP was measured using a cell counter. Noteworthy, a serial dilution of the platelet was used in the counting process to avoid aggregation in the cell counter. Platelet-derived biomaterial (PdB) was prepared by freezing and thawing platelet to disrupt the platelets membrane and to release its internal contents. For this purpose, prepared PRP was stored at −70 °C for 30 minutes then thawed in a 37 °C water bath for another 30 minutes. The freezing and thawing stage was repeated for 4 times. Finally, the suspension was centrifuged at 300 rpm for 15 minutes to remove the platelet lysate bodies. The supernatant, “freeze- PdB” (F-PdB), which was free of the platelet bodies was separated using a Pasteur pipette. Using CaCl_2_ as a platelet activator was another way to prepare CaCl_2_-PdB (CaCl_2_-PdB) in this study. For this purpose, PRP which was prepared in the prior stage was stored at 37 °C for 30 minutes then an equal volume of CaCl_2_ (25 millimolar) was added to activate the platelets. Finally, the suspension was centrifuged at 300 rpm for 15 minutes to remove the platelets remaining bodies and the supernatant which was free of the platelet bodies called “CaCl_2_-PdB” was separated using a Pasteur pipette^35,36^.

### Bacterial strains and growth conditions

*P. aeruginosa* and MRSA were collected from patients’ wounds and the isolates were grown in brain heart infusion (BHI) broth medium overnight. Then, the bacteria were transferred to a fresh culture medium and incubated at 37 °C until reaching the mid-logarithmic phase. The subcultures were centrifuged at 1,000 g for 15 min, the supernatant was discarded and the bacterial pellet was washed twice with sterile saline and resuspended in cold PBS to achieve a concentration of approximately 10^8^ CFU/ml. The bacterial suspension was kept on ice until further use^37,38^.

### Bacteria growth inhibition by disk diffusion method

Both isolates grown in agar were dissolved in sterile saline solution and the spectrophotometer measurement of the solutions at 600 nm showed a light absorption value of 0.3 units which indicated more than 10^8^ CFU/ml of the bacteria in the solution. After making bacteria stock solutions, both bacteria were cultured on a Muller Hinton agar (MHA) medium, afterward, disks soaked with approximately 30 µL of PdB (F-PdB and CaCl_2_-PdB) and plasma (as the control) were placed on the MHA medium. The plates were incubated overnight at 37 °C and the results of growth inhibition surrounding the discs observed visually. The size of the inhibition zone was rated based on the following criteria: no inhibition zone, inhibition zone of 0.5, 0.5–1, and greater than 1 mm^21,37^.

### Determination of antibacterial activity by microdilution broth

The minimum inhibitory concentration (MIC) of PdB was determined by the standard broth microdilution method using Mueller-Hinton broth (MHB) and custom- made 96-well plates. After seeding bacteria in trypticase soy agar, for each isolates a suspension of bacteria in the growth medium of MHB was prepared with an optical density equal to 0.5 McFarland (1 × 10^8^ CFU/mL). Using proper dilution, a concentration of 1 × 10^5^ CFU/mL was prepared. Afterward, 10 μl of each suspension was poured into a 96-wells microplate containing 100 μl of MHB and serial 2-fold dilution (ranging from 1:2 to 1:2048) of each PdB. For the positive control, the bacterial suspension was inoculated in the wells containing MHB without PdB. The antimicrobial activity of plasma and CaCl_2_ was also measured. After incubation at 37 °C for 24 hours, the MIC values were determined as the lowest dilution in which no bacterial growth is visible. The test was performed in duplicate for each isolate and the test was repeated in case the MIC values for the two wells of each PdB differed^39^.

### Time-kill assay

This method was performed in duplicate, by inoculating 5 × 10^5^ (CFU/mL) of mid-logarithmic-phase bacterial cells into 2 mL of MHB. Immediately after platelets activation, 250 µL from PdB or the same volume of 0.9% saline and plasma (normal control) were added into the tubes containing 250 µL of MHB inoculated with the relevant strains. Cultures were incubated at 37 °C with shaking (200 rpm) and 50 µL of samples were removed at 0, 4 and 8 hours after the inoculation stage for bacterial counts. Then, each sample serially diluted 10-fold in 0.9% saline, then 10 µL of each sample was spread onto agar plates. Bacterial colonies were counted (CFU/mL) and repeated measures of the Kruskal-Wallis test were performed to determine the differences between the variables^20^.

### Burn procedure

Forty-eight young male Wistar albino rats weighing 250–300 grams were divided randomly into four groups as follows: control group (n = 12) which was exposed to burn injury then infected by MRSA but received no treatment, F-PdB and CaCl_2_-PdB treated animals (n = 12, in each group) and the last group which was treated with plasma (n = 12). The animals were obtained 1 week before the experiment and kept in appropriate cages under controlled temperature (22 °C), humidity, lighting conditions (12-hour light/dark cycle), and had unrestricted access to food and water. Dorsal hair of rats was shaved with electric clippers and the rats were anesthetized by an intraperitoneal injection of 50 mg/kg ketamine hydrochloride and 10 mg/kg xylazine hydrochloride. Full-thickness, 5-mm diameter burn injury sites were generated using a calibrated bar with a temperature of 160 °C. The calibrated bar was applied to the dorsal shaved skin of the rats for 10 seconds (for both of the control and PdB–treated groups). Then, a suspension containing 5 × 10^8^ MRSA cells was inoculated onto each wound and 24 hours after infection, 100 μl of each PdB, plasma, and saline were injected separately inside the infected lesions sites (each bio-material was injected into the lesion created in an animal) for 14 days. It should be noted that PdBs were injected every day due to the short lifetime of platelets. After experimentation, all rats were kept in special cages under controlled temperature and were fed with standard rat chow and water. Wound tissues were excised on days 3 and 7, homogenized in 10 ml PBS, serially diluted, and plated onto the MHA. Finally, the colony-forming units (CFUs) were quantified and analyzed for statistical significance using the Kruskal-Wallis test. Moreover, photographs were taken just after the burn induction and 4, 7 and 14 days later without removing the scab.

### Measurement

On day 14, rats were euthanized under deep anesthesia (60 mg/kg intraperitoneal ketamine) and a fragment of the wound was excised and fixed in 10% formalin and paraffin-embedded. Afterward, the tissues were cut on a microtome into 4-micron vertical sections and were stained with hematoxylin and eosin (H&E). Finally, the wound healing hallmarks were measured using a light microscope; angiogenesis, polymorphonuclear leucocytes (PMNL), and re-epithelialization were observed and the images were captured without further processing^15,25^.

### Indirect immunofluorescence

Rat skin samples (from four groups) were fixed in 4% neutral buffered formaldehyde, embedded in paraffin then cut on a microtome into 5 µm sections. The sections were mounted on glass slides coated with APES (Aminopropyl-tri-ethoxy-silane). After deparaffinization and rehydration of the slides using a range of xylene and graded ethanol, antigen retrieval was performed by boiling the slides in sodium citrate buffer (pH 6.0) and maintained at a sub-boiling temperature for 10 minutes using a microwave. After cooling the slides on the bench-top for 30 minutes the tissue sections were embedded in 3.0% hydrogen peroxide in methanol for 15 minutes to block endogenous peroxidase activity. To permeabilize the tissue sections they were preincubated with 1% bovine serum albumin (BSA) and 0.3% Triton X-100 in PBS at room temperature for 15 min. Subsequently, the sections were incubated for 2 hours at room temperature with the primary antibody (rabbit anti-collagen I antibody, diluted in PBS 1:100). After washing the slides with PBS for 10 minutes the tissue sections were incubated for another 2 hours at room temperature with the secondary antibody (Goat anti-rabbit IgG FITC, diluted in PBS 1:150). The stained sections were mounted with prolong gold antifade, containing DAPI for nuclear stain. Finally, the samples were assessed using a Labomed Lx400 fluorescent microscope equipped with an Olympus color view III camera^40^.
